# Acute Kidney Injury After Cardiac Surgery in Low- and Middle-Income Countries

**DOI:** 10.5334/aogh.5185

**Published:** 2026-07-27

**Authors:** Emmanuel Ndaba, Vukosi Baloyi

**Affiliations:** 1 Department of Surgery, School of Clinical Medicine, University of the Witwatersrand, Johannesburg, South Africa

**Keywords:** acute kidney injury, cardiac surgery, cardiopulmonary bypass, renal replacement therapy, perioperative care, global cardiac anesthesia

## Abstract

*Background:* Cardiac-surgery-associated acute kidney injury (CSA-AKI) is a frequent postoperative complication worldwide and is associated with increased mortality, prolonged intensive care unit stay, and substantial resource utilization. While reported incidence appears broadly similar across high-income countries (HICs) and low- and middle-income countries (LMICs), emerging evidence suggests marked disparities in severity at presentation and clinical outcomes.

*Objectives:* To compare the epidemiology, severity, outcomes, and health-system determinants of CSA-AKI across HIC and LMIC settings, and to identify system-level contributors to excess mortality in resource-limited environments.

*Methods:* We conducted a focused narrative review of contemporary observational studies reporting CSA-AKI incidence, severity, renal replacement therapy (RRT) utilization, and mortality following adult cardiac surgery. Literature was identified through targeted searches of PubMed/MEDLINE and hand-searching of reference lists. Priority was given to studies using standardized AKI definitions (RIFLE, AKIN, or KDIGO) and reporting clinically relevant perioperative outcomes.

*Findings:* Across income settings, CSA-AKI incidence ranged from approximately 20% to 43%. However, LMIC cohorts consistently demonstrated more advanced AKI at diagnosis and substantially higher AKI-associated mortality, particularly among patients requiring RRT. In HICs, dialysis-requiring CSA-AKI was uncommon (~1%) but carried high mortality despite early detection and unrestricted access to renal support. In contrast, LMIC settings reported lower RRT utilization, delayed initiation, and mortality exceeding 40–55% in advanced AKI stages.

*Conclusions:* CSA-AKI is a common global complication of cardiac surgery with disproportionately severe consequences in resource-limited settings. Excess mortality in LMICs appears largely driven by delayed detection and constrained rescue capacity rather than biological susceptibility alone. Strengthening perioperative surveillance and access to timely renal support may substantially reduce avoidable mortality.

## Introduction

Acute kidney injury (AKI) remains one of the most common and clinically consequential complications following adult cardiac surgery, affecting between 20% and 40% of patients depending on case mix, definition applied, and intensity of postoperative surveillance [1–3]. Even modest postoperative increases in serum creatinine are independently associated with increased short- and long-term mortality, prolonged intensive care unit (ICU) stay, higher rates of renal replacement therapy (RRT), and substantial healthcare costs [4–6]. Despite advances in surgical technique, cardiopulmonary bypass (CPB) technology, and perioperative critical care, the global burden of cardiac-surgery-associated AKI (CSA-AKI) has remained largely unchanged over the past two decades [2,7].

AKI is uniquely suited as a lens through which to examine disparities between high-income countries (HICs) and low- and middle-income countries (LMICs). Unlike many postoperative complications, AKI is objectively defined, routinely measured, and strongly prognostic across diverse healthcare systems. Standardized classification systems—particularly those proposed by the Kidney Disease: Improving Global Outcomes (KDIGO)—permit meaningful comparisons across regions with markedly different patient populations, surgical volumes, and resource availability [8]. As a result, AKI provides a rare opportunity to move beyond anecdotal descriptions of inequity toward measurable, outcome-linked comparisons between global cardiac surgery systems.

Importantly, emerging evidence suggests that the excess mortality associated with CSA-AKI in LMICs is not solely attributable to biological susceptibility or surgical complexity. Rather, differences in preoperative risk optimization, intraoperative monitoring, postoperative surveillance, and—critically—access to timely RRT appear to play a dominant role [9–11]. In this context, AKI functions less as an isolated renal complication and more as a sentinel marker of system capacity, reflecting the ability of healthcare structures to detect injury early, prevent progression, and rescue organ dysfunction once it occurs.

The objective of this review is, therefore, to synthesize contemporary evidence on CSA-AKI with a specific focus on contrasting HIC and LMIC settings. We aim to (i) summarize global epidemiology using harmonized definitions, (ii) highlight system-level contributors to observed outcome disparities, and (iii) identify pragmatic, context-appropriate strategies to reduce the burden of CSA-AKI in resource-constrained environments.

## Methods

This manuscript was conducted as a focused narrative review and was not designed as a systematic review or meta-analysis. The objective was to synthesize clinically relevant evidence comparing the epidemiology, severity, outcomes, and health-system determinants of CSA-AKI between HICs and LMICs, with an emphasis on factors influencing perioperative detection, management, and rescue capacity.

Relevant literature was identified through targeted searches of PubMed/MEDLINE, supplemented by hand-searching reference lists from key guidelines, consensus statements, and landmark cohort studies. Search terms included combinations of “acute kidney injury,” “cardiac surgery,” “cardiopulmonary bypass,” “renal replacement therapy,” “KDIGO,” “mortality,” “outcomes,” “low- and middle-income countries,” and “resource-limited settings.” The search strategy was intentionally pragmatic, prioritizing studies with direct clinical applicability to perioperative cardiac surgical practice.

Priority was given to studies reporting CSA-AKI using standardized definitions (RIFLE, AKIN, or KDIGO) and providing clinically meaningful outcomes, including mortality, RRT utilization, and length of ICU stay. Studies contributing to the core comparative evidence base are summarized in Table 1.

**Table 1:** Key studies on cardiac-surgery-associated acute kidney injury (CSA-AKI) in high-income and low-/middle-income settings.

STUDY (YEAR)	COUNTRY / REGION	INCOME SETTING	STUDY DESIGN	POPULATION	AKI DEFINITION	AKI INCIDENCE	SEVERE AKI / RRT	AKI-ASSOCIATED MORTALITY	KEY FINDINGS RELEVANT TO LMIC–HIC CONTRAST
**Hobson et al.** [4]	United States	HIC	Retrospective cohort	2,973 adult patients undergoing cardiothoracic surgery	RIFLE (serum creatinine–based)	~30%–43% (any AKI)	~1%–2% required RRT	Mortality increased stepwise with AKI severity; long-term mortality independently higher even after mild AKI	Demonstrates that even in high-resource settings with universal access to RRT, CSA-AKI—including mild forms—confers a durable survival disadvantage
**Thakar et al.** [6]	United States (Cleveland Clinic)	HIC	Retrospective cohort (derivation and validation)	33,217 adults undergoing open-heart cardiac surgery	Acute renal failure requiring dialysis	~1% overall (0.5%–22.1% across risk strata)	Dialysis-requiring AKI (primary outcome)	~40%–60% in patients requiring RRT	Establishes a high-resource benchmark: dialysis-requiring CSA-AKI is rare but carries extremely high mortality despite early detection and unrestricted RRT access
**Machado et al.** [5]	Brazil	Upper-middle-income (LMIC)	Retrospective cohort	2,804 adults undergoing cardiac surgery	KDIGO (serum creatinine only)	42% overall	2% required RRT (~65% of KDIGO stage 3)	30-day mortality rose sharply with AKI severity; 55% in KDIGO stage 3	High AKI incidence with limited RRT use; mortality markedly higher in severe AKI, highlighting vulnerability in resource-constrained settings
**Leballo et al.** [10]	South Africa	LMIC	Retrospective single-center cohort	476 adults undergoing cardiac surgery with cardiopulmonary bypass	KDIGO criteria	28% overall	~3% required RRT (subset of KDIGO stage 3)	In-hospital mortality: 21% with AKI vs 5% without; 44% in KDIGO stage 3	Demonstrates substantial mortality gradient by AKI severity in an LMIC setting with constrained access to advanced renal support
**Xie et al.** [9]	China	Upper-middle-income (LMIC)	Retrospective cohort	2,575 adults undergoing first cardiac surgery with CPB	KDIGO (serum creatinine only)	36% overall	1.2% required RRT	Mortality higher with AKI (2.6% vs 0.9%); RRT strongly associated with death (adjusted HR 18.68)	High AKI incidence with relatively low RRT utilization; mortality escalates dramatically once RRT is required

Findings are presented descriptively to highlight consistent patterns, contrasts, and system-level contributors to disparities in CSA-AKI outcomes across income settings, rather than to provide pooled quantitative estimates.

## Definitions and Clinical Relevance of AKI After Cardiac Surgery

Early studies of CSA-AKI were limited by heterogeneous definitions, ranging from absolute creatinine thresholds to subjective clinical judgment, resulting in substantial variability in reported incidence and outcomes. The introduction of consensus classification systems—RIFLE (Risk, Injury, Failure, Loss, End-stage kidney disease), AKIN (Acute Kidney Injury Network), and most recently KDIGO—represented a critical advance in standardizing AKI diagnosis and staging, enabling more reliable comparisons across studies and healthcare systems [8,12].

KDIGO criteria define AKI based on dynamic changes in serum creatinine and/or urine output, capturing the full spectrum of renal injury from mild, transient dysfunction to dialysis-requiring failure. This framework has become the dominant standard in cardiac surgery research and clinical practice and is endorsed by major professional societies, including the Society of Thoracic Surgeons and international anesthesia and perfusion groups [1,2]. Importantly, KDIGO staging demonstrates a graded, dose–response relationship with mortality after cardiac surgery, even at stage 1 disease, underscoring the clinical relevance of small postoperative creatinine rises that were previously considered benign [5,13].

From a prognostic standpoint, CSA-AKI is associated with a constellation of adverse outcomes, including increased in-hospital and long-term mortality, prolonged ICU and hospital length of stay, higher rates of mechanical ventilation, and substantial escalation in the cost of care [4–6]. Patients requiring RRT represent the extreme end of the disease spectrum and consistently experience mortality rates exceeding 40–60% in many series, particularly outside high-resource settings [10,11,14].

The timing of AKI recognition is increasingly recognized as a critical determinant of outcome. In HIC cardiac ICUs, structured urine output monitoring and routine daily creatinine surveillance facilitate early detection and prompt supportive interventions. In contrast, delayed recognition—often driven by limited laboratory access, staffing constraints, or competing clinical priorities—is common in LMIC settings and may permit progression from potentially reversible injury to established renal failure [9]. These differences in detection and response, rather than differences in AKI definition itself, are central to understanding global disparities in CSA-AKI outcomes.

## Global Epidemiology of Cardiac-Surgery-Associated AKI

### Incidence in High-Income Countries

Large registry-based and multicenter cohort studies from North America and Europe consistently report CSA-AKI incidence rates between 20% and 35% when contemporary KDIGO or RIFLE criteria are applied [2,3,7]. Data from high-volume centers demonstrate that while the overall incidence of AKI has remained relatively stable over time, the distribution is skewed toward milder stages, with only 1%–2% of patients progressing to dialysis-requiring AKI [2,3]. In the Cleveland Clinic cohort of over 33,000 cardiac surgical patients, dialysis-requiring CSA-AKI occurred in approximately 1% overall, though risk varied markedly across predefined strata, reaching >20% in the highest-risk patients [2].

Importantly, outcomes in HIC settings illustrate a dissociation between incidence and mortality. Despite stable AKI rates, AKI-associated mortality has declined, reflecting improvements in perioperative monitoring, early detection, and timely access to advanced renal support, including continuous renal replacement therapy (CRRT) [2,7]. Nevertheless, even in these high-resource environments, CSA-AKI remains prognostically significant. Cohort data demonstrate a graded, stepwise increase in both short- and long-term mortality across AKI severity, with excess mortality persisting even after apparent renal recovery [2,3]. These findings establish a critical benchmark: optimal detection and rescue reduce—but do not eliminate—the mortality burden of CSA-AKI.

### Incidence in Low- and Middle-Income Countries

In contrast, studies from LMIC settings consistently report higher CSA-AKI incidence, commonly ranging from 30% to over 40%, alongside a disproportionate burden of advanced disease at presentation [9–11,14–16]. Retrospective and prospective cohorts from Brazil, China, South Africa, India, and sub-Saharan Africa demonstrate not only higher overall AKI rates, but also a greater representation of KDIGO stage 2 and 3 disease, suggesting delayed recognition and limited perioperative surveillance [9–11,14–16].

Data from public-sector cardiac centers in South Africa and Brazil further highlight the downstream consequences of these patterns. Although rates of RRT remain low (approximately 1–3%), mortality among patients with severe CSA-AKI is strikingly high, with stage 3 AKI associated with in-hospital or 30-day mortality exceeding 40–55% in several cohorts [10,11]. This mirrors the mortality observed in dialysis-requiring AKI in HICs, but occurs in contexts where RRT access is constrained, initiation is delayed, and CRRT is often unavailable.

Direct comparisons between HIC and LMIC cohorts are complicated by heterogeneity in case mix, reporting practices, and monitoring intensity. However, a consistent epidemiologic signal emerges: patients in LMICs present later, with more severe renal injury, and experience substantially worse outcomes, even after accounting for comorbidity burden and surgical complexity [10,11]. Limited access to timely laboratory testing, reduced intraoperative hemodynamic monitoring, workforce constraints, and restricted critical care capacity likely contribute to both AKI progression and excess mortality.

## Preoperative Risk Profile: A Major LMIC Disadvantage

### Baseline renal reserve and comorbidity burden

Baseline renal vulnerability is a critical determinant of CSA-AKI risk, and substantial differences exist between patients undergoing cardiac surgery in HICs and LMICs. In HIC settings, structured preoperative assessment—including routine estimation of glomerular filtration rate (eGFR), optimization of volume status, and medication review—allows identification and mitigation of renal risk prior to surgery [1,2]. In contrast, cohorts from LMICs consistently demonstrate a high prevalence of unrecognized or underdocumented chronic kidney disease (CKD), hypertension, and diabetes mellitus at the time of surgical admission, reflecting broader gaps in primary and secondary preventive care [11,17,18].

Data summarized in Table 1 illustrate that LMIC cardiac surgery cohorts report AKI incidences ranging from approximately 28% to over 40%, with a disproportionate burden of KDIGO stage 2–3 disease at diagnosis, compared with predominantly stage 1 presentations in HIC cohorts. Public-sector studies from India and sub-Saharan Africa further demonstrate that impaired baseline renal function is frequently identified only during preoperative admission rather than through longitudinal outpatient follow-up [10]. This diminished renal reserve reduces physiological tolerance to perioperative hemodynamic stress and lowers the threshold for progression from transient creatinine elevation to established AKI. Importantly, these baseline differences are rarely captured in risk prediction models derived from HIC populations, contributing to systematic underestimation of AKI risk in LMIC patients.

### Late surgical referral and advanced cardiac disease

Late referral for definitive cardiac surgery further compounds renal vulnerability in LMIC settings. Patients frequently present with advanced valvular disease, severe ventricular dysfunction, and chronic congestive states, all of which are independently associated with renal hypoperfusion and venous congestion [9,19,20]. Prolonged exposure to low cardiac output and elevated central venous pressure results in subclinical renal injury even before the operative insult occurs, effectively narrowing the margin for perioperative renal resilience.

Evidence from South African and Indian cardiac surgery cohorts demonstrates that many patients arrive for surgery already in a state of pre-existing cardiorenal stress, rendering them particularly susceptible to CSA-AKI despite technically successful procedures [10]. As reflected in Table 1, these cohorts not only exhibit higher AKI incidence but also markedly higher AKI-associated mortality, especially among patients progressing to KDIGO stage 3 disease. In this context, postoperative AKI often represents the culmination of a chronic pathophysiological trajectory rather than an isolated perioperative complication.

## Intraoperative Factors and Resource Constraints

### Cardiopulmonary bypass exposure and perfusion strategies

CPB remains a central contributor to CSA-AKI through mechanisms including non-pulsatile flow, hemodilution, systemic inflammatory activation, and renal hypoperfusion. In HIC centers, advances in perfusion technology—such as goal-directed perfusion, higher indexed pump flow targets, tighter hematocrit control, and near-continuous monitoring of perfusion indices—have been increasingly adopted to mitigate renal risk during CPB [1,21]. Registry-based and multicenter cohort data from these settings demonstrate that, despite stable AKI incidence over time, progression to severe AKI and dialysis-requiring renal failure has declined in parallel with improvements in intraoperative perfusion and rescue strategies.

In contrast, data from LMIC cardiac surgery cohorts summarized in Table 1 consistently demonstrate higher proportions of moderate-to-severe AKI at diagnosis, suggesting greater cumulative intraoperative renal insult. Longer CPB times are commonly reported in these settings, reflecting greater disease complexity at presentation, workflow inefficiencies, and limited access to minimally invasive or off-pump techniques [9,10,22]. Studies from Brazil, China, South Africa, and India report AKI incidences ranging from approximately 28% to over 40%, with KDIGO stage 2–3 disease accounting for a substantial proportion of cases—patterns that are closely aligned with prolonged CPB exposure and limited intraoperative renal protection. Additionally, constraints on disposables, perfusion equipment, and staffing may limit implementation of renal-protective strategies that are routine in high-resource environments, amplifying susceptibility to CPB-related injury.

### Intraoperative monitoring limitations

Continuous hemodynamic monitoring plays a critical role in preventing renal hypoperfusion during cardiac surgery. In HIC operating theatres, invasive arterial pressure monitoring, central venous pressure trend analysis, and—in selected centers—cerebral or renal near-infrared spectroscopy (NIRS) are routinely used to guide perfusion and vasoactive therapy in real time [21,23]. These approaches facilitate early detection of occult hypotension, low cardiac output states, and perfusion–pressure mismatch, allowing timely corrective interventions that limit cumulative renal injury.

By contrast, intraoperative monitoring in many LMIC cardiac centers relies on intermittent measurements with limited physiological granularity. Advanced adjuncts such as renal oximetry are rarely available, and perfusion targets may be guided by population-based norms rather than patient-specific physiology. The tabled evidence demonstrates that LMIC cohorts not only experience higher AKI incidence but also a marked escalation in mortality once severe AKI develops, particularly among patients requiring RRT. This pattern is consistent with unrecognized or inadequately corrected episodes of intraoperative renal hypoperfusion, which accumulate over prolonged CPB runs and manifest postoperatively as advanced AKI.

## Postoperative Detection and Diagnostic Delays

### Timing of AKI recognition

Early recognition of AKI is a cornerstone of renal-protective care following cardiac surgery. In HIC cardiac ICUs, standardized postoperative pathways incorporating frequent serum creatinine measurement urine output monitoring, and implementation of KDIGO-based care bundles enable timely diagnosis and early supportive intervention [2,24]. These protocols facilitate early hemodynamic optimization, avoidance of nephrotoxins, adjustment of fluid and vasoactive therapy, and early nephrology consultation, contributing to improved renal recovery and reduced progression to dialysis-requiring AKI.

In contrast, data synthesized in Table 1 demonstrate that postoperative AKI in LMIC settings is frequently recognized later in its clinical course. Limited laboratory availability, reduced testing frequency, batching of samples, and staffing constraints commonly delay detection until overt oliguria or marked creatinine elevation has occurred [7,10]. This delayed recognition aligns with the consistently higher representation of KDIGO stage 2–3 disease and dialysis-requiring AKI reported across LMIC cardiac surgery cohorts.

The tabled evidence further indicates that this delay in recognition is not merely diagnostic but translates directly into worse outcomes. LMIC studies consistently report higher AKI-attributable mortality compared with HIC cohorts, even when overall AKI incidence is comparable. These findings suggest that the timing of detection—rather than AKI occurrence alone—is a critical determinant of prognosis following cardiac surgery.

### Biomarkers and early detection tools

Novel biomarkers such as neutrophil gelatinase-associated lipocalin (NGAL) and cystatin C have shown promise for early AKI detection in cardiac surgery populations, particularly in HIC research settings [25,26]. These biomarkers may identify renal injury hours to days before creatinine rises, theoretically expanding the window for preventive intervention.

However, as reflected in Table 2, these assays remain largely unavailable in LMIC clinical practice due to cost, infrastructure requirements, and limited laboratory capacity. As a result, clinicians in resource-constrained environments continue to rely almost exclusively on serum creatinine—a late and insensitive marker of renal injury. This dependence reinforces diagnostic delay, particularly in the immediate postoperative period when renal injury may already be evolving despite preserved urine output.

**Table 2:** LMIC-Specific Health-System Challenges and Their Clinical Consequences in Cardiac Surgery–Associated Acute Kidney Injury (CSA-AKI).

HEALTH-SYSTEM CHALLENGE (LMIC)	MECHANISM OF IMPACT	CLINICAL CONSEQUENCE IN CSA-AKI	CONTRAST WITH HIC SETTINGS
**Late referral for cardiac surgery**	Prolonged exposure to low cardiac output, venous congestion, and neurohormonal activation before surgery	Reduced baseline renal reserve; higher susceptibility to perioperative AKI; advanced AKI at diagnosis	Earlier referral and elective optimisation reduce preoperative renal stress
**High burden of undiagnosed CKD and comorbidities**	Limited primary care screening; poor chronic disease control	AKI occurs on a background of chronic renal vulnerability, accelerating progression to severe stages	Routine CKD detection and optimisation common preoperatively
**Limited preoperative optimisation**	Short preoperative admission windows; lack of multidisciplinary assessment	Inadequate volume, blood pressure, and medication optimisation before CPB	Structured prehabilitation and risk stratification pathways
**Longer cardiopulmonary bypass times**	Case complexity, workflow inefficiencies, limited access to off-pump techniques	Greater inflammatory burden, renal hypoperfusion, and haemodilution	Shorter CPB duration and goal-directed perfusion strategies
**Reduced intraoperative monitoring capacity**	Reliance on intermittent haemodynamic measurements; absence of renal/cerebral oximetry	Occult hypotension and renal hypoperfusion go undetected	Continuous invasive monitoring and perfusion-guided targets
**Delayed postoperative laboratory testing**	Limited lab availability, staffing shortages, batching of samples	AKI recognised at later, less reversible stages	Early creatinine trends and urine output protocols
**Absence of early AKI biomarkers**	Cost and infrastructure constraints	Reliance on late creatinine rise; missed therapeutic window	NGAL, cystatin C used selectively for early detection
**Limited nephrology availability**	Workforce shortages; competing service demands	Delayed specialist input and delayed RRT decision-making	Early nephrology consultation integrated into ICU care
**Restricted access to renal replacement therapy (RRT)**	Limited dialysis machines; prioritisation pressures; cost barriers	Dialysis-requiring AKI becomes a high-mortality phenotype	CRRT readily available; early initiation feasible
**Predominant use of intermittent haemodialysis**	Infrastructure limitations	Poor haemodynamic tolerance in vasoplegic post-cardiac surgery patients	CRRT preferred for unstable patients
**Absence of AKI prevention bundles**	Lack of protocolised care pathways	Inconsistent fluid, drug, and monitoring practices	Standardised AKI bundles and quality metrics
**Lack of national cardiac surgery registries**	Limited data capture and feedback loops	Under-recognition of AKI burden; limited quality improvement	Continuous benchmarking and outcome monitoring

The clinical consequence of this delay is a narrowing of the therapeutic window in which AKI may be reversible. By the time creatinine rises are detected, renal injury may already be established, limiting the effectiveness of supportive measures and increasing the likelihood of progression to dialysis-requiring failure. This pattern is consistently reflected in the tabled LMIC cohorts, where delayed detection is closely coupled with higher rates of severe AKI, limited renal recovery, and excess mortality.

## Access to Renal Replacement Therapy: The Core LMIC Challenge

### Availability of renal replacement therapy

Among patients who develop severe CSA-AKI, access to timely RRT represents the single most important determinant of survival. In high-income settings, escalation to RRT—most commonly in the form of CRRT—is routinely available within cardiac ICUs, supported by trained staff, dedicated machines, and established protocols for early initiation [1,2,27]. As reflected in the studies summarized in Table 1, this capacity allows for hemodynamic stabilization, controlled fluid balance, and metabolic support during periods of cardiac and renal instability, attenuating progression from severe AKI to irreversible multiorgan failure.

In contrast, Table 2 highlights that access to RRT in LMICs is frequently constrained by structural, logistical, and economic limitations. Public-sector hospitals in sub-Saharan Africa and South Asia report limited dialysis capacity that is often shared across multiple ICUs and medical wards, resulting in prioritization driven by bed availability, staffing, and affordability rather than clinical indication alone [10,11,18]. In this context, patients with CSA-AKI may experience prolonged delays before dialysis initiation or may not receive RRT at all despite meeting accepted criteria.

The tabled evidence further demonstrates that modality availability differs substantially between settings. While CRRT is the dominant modality in HIC cardiac ICUs, LMIC centers rely predominantly on intermittent hemodialysis, often delivered in unstable postoperative patients due to infrastructure limitations. This mismatch between patient physiology and dialysis modality contributes to poor hemodynamic tolerance and limits the ability to provide sustained renal support during periods of vasoplegia or low cardiac output, as consistently noted across LMIC cohorts.

Crucially, outcome data summarized in Table 1 show that dialysis-requiring CSA-AKI in LMICs is associated with disproportionately high mortality, frequently exceeding 50%, particularly in public-sector cardiac surgery programs [10]. These mortality rates contrast sharply with those reported in HIC cohorts, where earlier initiation, continuous modalities, and integrated critical care support mitigate—but do not eliminate—the lethality of severe AKI. The convergence of delayed initiation, intermittent therapy, and constrained rescue capacity positions dialysis-requiring CSA-AKI as a high-mortality phenotype in LMIC settings.

Taken together, these findings indicate that differences in rescue capacity, rather than AKI incidence alone, drive much of the observed mortality gap between LMICs and HICs. Access to timely, appropriate RRT emerges from the tabulated data as the central determinant of survival once severe CSA-AKI has developed, underscoring the role of AKI as a marker of health-system performance rather than an isolated postoperative complication.

### Timing and modality of RRT

Beyond availability, the timing and modality of RRT differ substantially between healthcare settings, with important implications for outcomes after cardiac surgery. In HIC cardiac ICUs, CRRT is favored for hemodynamically unstable postoperative patients, allowing gradual solute clearance, precise fluid balance, and avoidance of abrupt intravascular shifts [2,27]. Early initiation strategies—particularly in the context of fluid overload, refractory metabolic derangements, or evolving multiorgan dysfunction—are facilitated by protocolized care pathways and dedicated resources, and have been associated with improved hemodynamic tolerance and, in selected studies, better outcomes [14].

In LMICs, intermittent hemodialysis remains the predominant RRT modality due to lower cost and reduced infrastructure requirements. However, intermittent therapy is frequently poorly tolerated in vasoplegic or low-output states common after cardiac surgery, resulting in intradialytic hypotension, treatment interruption, or suboptimal solute and fluid clearance [18]. These limitations are compounded by delayed initiation, as patients often reach thresholds for RRT only after marked metabolic derangement and established multiorgan dysfunction, by which time physiological reserve for recovery is substantially reduced.

The tabulated evidence highlights that differences in modality selection are closely linked to differences in timing. In HIC cohorts, continuous modalities permit earlier escalation of renal support during evolving injury, whereas in LMIC settings, initiation of RRT is frequently deferred until overt, late-stage indications emerge, reflecting constrained capacity and prioritization pressures. As a result, dialysis initiation in LMIC cardiac ICUs commonly occurs later in the disease trajectory, when renal injury is less reversible and systemic complications are already established.

Critically, mortality differences between HIC and LMIC cardiac surgery populations appear to be driven less by how often AKI occurs and more by whether patients with severe AKI can be effectively rescued once injury has developed. The convergence of delayed initiation, reliance on intermittent modalities, and limited ability to deliver sustained renal support positions dialysis-requiring CSA-AKI as a disproportionately lethal complication in resource-constrained settings.

## Outcomes: Mortality, Length of Stay, and Resource Utilization

### AKI-attributable mortality

Across all healthcare settings, CSA-AKI is independently associated with increased mortality in a graded fashion according to severity. Large HIC cohorts consistently demonstrate stepwise increases in both in-hospital and long-term mortality with each advancing AKI stage, even after adjustment for baseline comorbidity burden and operative complexity [3,6,13]. Importantly, improvements in perioperative surveillance, hemodynamic management, and access to advanced critical care have translated into improved survival among patients with mild to moderate AKI in high-resource environments over time.

In contrast, mortality associated with CSA-AKI in LMIC settings remains substantially higher at comparable stages of renal injury. Cohort studies from Brazil, India, China, and South Africa consistently report elevated case-fatality rates per AKI stage, with dialysis-requiring AKI emerging as a particularly lethal phenotype [5,9–11,15]. Across these settings, mortality frequently exceeds 40–50% once RRT is required, a pattern that persists even when standardized definitions such as KDIGO are applied. This divergence highlights that outcome disparities are driven predominantly by post-diagnostic care capacity—including delayed detection, limited monitoring, and restricted access to sustained renal support—rather than by differences in diagnostic classification alone.

The tabulated data further demonstrate that while AKI incidence may be broadly similar across income settings, progression to advanced stages and death occurs more frequently in LMIC cohorts. These findings support the concept of CSA-AKI as a condition in which health-system responsiveness, rather than biological susceptibility, is the dominant determinant of survival once injury has occurred.

### Length of stay and opportunity cost

CSA-AKI is also associated with prolonged ICU and hospital length of stay across all healthcare systems. In HICs, this increase in length of stay primarily translates into higher resource utilization and cost but is often accommodated within well-resourced critical care infrastructures, supported by step-down facilities and established discharge pathways [6,13]. Consequently, prolonged admission does not necessarily translate into system-level congestion or access limitation.

In LMIC settings, however, prolonged ICU occupancy by patients with CSA-AKI carries a substantial opportunity cost. Data from public-sector hospitals demonstrate that delayed renal recovery, limited access to step-down care, and slow escalation to definitive therapies—particularly RRT—result in disproportionately long ICU stays despite overall resource scarcity [10,18]. This paradox, whereby patients remain in high-acuity beds longer despite fewer available resources, exacerbates existing bed shortages and constrains access for other critically ill surgical and medical patients.

The tabulated evidence underscores that prolonged length of stay in LMICs is not simply a marker of illness severity but reflects systemic inefficiencies in escalation, rescue, and recovery pathways. As a result, the burden of CSA-AKI in resource-limited settings extends beyond individual patient outcomes to exert a measurable strain on already constrained critical care systems.

## Health-System and Structural Barriers in LMICs

The excess burden of CSA-AKI in LMICs cannot be fully explained by patient-level risk factors or surgical complexity alone. Rather, it reflects a convergence of health-system limitations that impair prevention, detection, and rescue across the perioperative continuum. Workforce shortages—particularly of trained ICU nurses, perfusionists, and nephrologists—limit the ability to deliver continuous monitoring, timely escalation of care, and sustained renal support [16,28]. In many LMIC centers, competing demands for critical care beds further constrain postoperative surveillance, often necessitating early transfer from ICU despite ongoing organ dysfunction.

Financial toxicity represents an additional and often under-recognized barrier. In many LMIC settings, the cost of prolonged ICU care and RRT is borne partially or entirely by patients and their families, directly influencing decisions regarding escalation, modality, and duration of therapy [18,28]. These economic pressures may result in delayed initiation of RRT, premature discontinuation, or non-initiation despite accepted clinical indications. The absence of structured AKI prevention bundles and national cardiac surgery registries further limits quality-improvement efforts and obscures the true magnitude of CSA-AKI-associated morbidity and mortality.

Key argument: outcomes following CSA-AKI reflect system capacity—encompassing surveillance infrastructure, workforce availability, and rescue capability—rather than patient biology alone. Addressing AKI in LMICs, therefore, requires system-level interventions that extend beyond the operating theatre.

## Strategies to Reduce the Burden of CSA-AKI in LMIC Settings

Reducing the burden of CSA-AKI in LMICs requires strategies that are context-appropriate, scalable, and cost-conscious, rather than wholesale transplantation of HIC technologies. Given that much of the excess mortality appears to arise from delayed detection and limited rescue capacity, interventions with the greatest potential impact are those that improve early recognition, standardise basic care, and optimize use of existing resources.

## Low-Cost, High-Yield Interventions

Several interventions with strong biological plausibility and supportive evidence can be implemented with minimal infrastructure investment. protocolized perioperative fluid management—avoiding both hypovolemia and fluid overload—represents a foundational strategy. In LMIC settings, where advanced hemodynamic monitoring may be unavailable, structured clinical assessment combined with simple dynamic indices can meaningfully reduce renal hypoperfusion risk.

Avoidance of nephrotoxins—including non-steroidal anti-inflammatory drugs, unnecessary contrast exposure, and inappropriate antibiotic dosing—remains a critical but often overlooked preventive measure. Standardization of serum creatinine and urine output monitoring, even at reduced testing frequency, can facilitate earlier identification of evolving AKI when combined with predefined escalation triggers. Importantly, these measures do not require new technology but rather protocol discipline, staff education, and institutional prioritization of AKI as a preventable complication.

## Context-Appropriate Prevention Bundles

Adaptation of existing AKI prevention frameworks, such as those proposed by KDIGO, into simplified, locally feasible bundles offers a pragmatic path forward. In LMIC cardiac surgery units, such bundles may include structured preoperative identification of high-risk patients using baseline creatinine and comorbidity profiles, intraoperative targets for blood pressure and perfusion time, and postoperative checklists mandating urine output documentation and daily creatinine review for the first 72 hours.

Early involvement of nephrology services, where available, has been associated with improved outcomes in AKI and may be particularly valuable in LMIC settings where delayed escalation is common. Even in the absence of on-site nephrologists, telemedicine consultation, standardized referral criteria, or trigger-based escalation pathways may help bridge expertise gaps and reduce delays in decision-making.

## Research and Policy Priorities

Sustainable improvement in CSA-AKI outcomes will require parallel investment in data systems and implementation science. Development of LMIC-specific cardiac surgery registries incorporating standardized AKI definitions and outcomes would enable benchmarking, quality improvement, and more accurate estimation of disease burden. Pragmatic clinical trials focusing on low-cost interventions—rather than expensive technologies—are more likely to yield actionable results in these settings.

At a policy level, prioritization of critical care capacity, dialysis access, and workforce training should be viewed as integral components of cardiac surgery programs rather than ancillary services. Strengthening these systems may yield benefits that extend beyond renal outcomes, improving overall postoperative survival and resource efficiency.

## Future Directions and Research Gaps

Despite growing recognition of CSA-AKI as a global problem, substantial gaps remain in the evidence base, particularly in LMIC contexts. There is a pressing need for multicenter LMIC cohorts using harmonized KDIGO definitions to accurately characterize incidence, severity, and outcomes. Current data are often derived from single-center studies with limited generalizability and inconsistent follow-up.

Standardized reporting of AKI timing, access to RRT, and cause-specific mortality would enhance comparability across regions. Additionally, existing risk prediction models—largely developed in HIC populations—require validation and recalibration for LMIC settings, where baseline risk profiles and system constraints differ markedly.

Implementation science offers an underutilized but highly relevant framework for AKI research in resource-limited environments. Understanding how evidence-based practices can be effectively integrated into overstretched health systems may prove more impactful than further refinement of biological mechanisms or biomarkers that remain inaccessible to most LMIC centers.

## Conclusion

CSA-AKI remains a common complication across all healthcare systems, but its consequences are profoundly unequal. While the incidence of CSA-AKI is broadly comparable between HICs and LMICs, outcomes diverge sharply once injury occurs. This review demonstrates that excess mortality in LMIC settings is driven less by differences in biological susceptibility or surgical complexity and more by systemic limitations that delay detection, constrain perioperative optimization, and restrict access to timely RRT.

Across the perioperative continuum, patients in LMICs enter surgery with reduced renal reserve, experience longer CPB exposure and less granular intraoperative monitoring, and are diagnosed with AKI later in its clinical course. Once severe AKI develops, limited dialysis capacity, delayed initiation, and reliance on hemodynamically poorly tolerated modalities amplify mortality risk. In contrast, improvements in surveillance, perfusion strategies, and rescue capacity in high-income settings have translated into declining AKI-associated mortality despite stable incidence.

These findings underscore the importance of reframing CSA-AKI not merely as a postoperative renal complication, but as a sentinel marker of health-system performance in cardiac surgery. Addressing this disparity will require context-appropriate strategies focused on early recognition, standardized basic care, and expansion of critical care and dialysis capacity rather than wholesale adoption of high-cost technologies. For clinicians, anesthesiologists, perfusionists, and policymakers alike, improving outcomes after CSA-AKI in resource-limited settings represents an opportunity to deliver high-impact gains in perioperative survival through system-level optimization rather than biological innovation alone.
